# Link prediction in complex networks via matrix perturbation and decomposition

**DOI:** 10.1038/s41598-017-14847-2

**Published:** 2017-11-07

**Authors:** Xiaoya Xu, Bo Liu, Jianshe Wu, Licheng Jiao

**Affiliations:** Key Laboratory of Intelligent Perception and Image Understanding of Ministry of Education, International Research Center for Intelligent Perception and Computation, Joint International Research Laboratory of Intelligent Perception and Computation, Xidian University, Xi’an, Shaanxi Province 710071 China

## Abstract

Link prediction in complex networks aims at predicting the missing links from available datasets which are always incomplete and subject to interfering noises. To obtain high prediction accuracy one should try to complete the missing information and at the same time eliminate the interfering noise from the datasets. Given that the global topological information of the networks can be exploited by the adjacent matrix, the missing information can be completed by generalizing the observed structure according to some consistency rule, and the noise can be eliminated by some proper decomposition techniques. Recently, two related works have been done that focused on each of the individual aspect and obtained satisfactory performances. Motivated by their complementary nature, here we proposed a new link prediction method that combines them together. Moreover, by extracting the symmetric part of the adjacent matrix, we also generalized the original perturbation method and extended our new method to weighted directed networks. Experimental studies on real networks from disparate fields indicate that the prediction accuracy of our method was considerably improved compared with either of the individual method as well as some other typical local indices.

## Introduction

Link prediction aims at revealing missing or potential relations between data entries from large volumes of data sets which are subject to dynamical changes and uncertainty. With the rapid development of studies on complex networks, the problem of link prediction has received extensive attention from researchers in various fields including physics, mathematics, computer science, social science and so on^1,2^. On the one hand, the investigation of complex networks may provide some novel insights on real-world linking patterns which is helpful to link prediction. On the other hand, by trying to predict the missing or potential links with a high accuracy, link prediction may also help to provide us with a deeper understanding of the organization of real world networks, which is a longstanding challenge in many branches of science^3^. From a practical point of view, link prediction is also a fundamental issue for many modern world applications in disparate fields such as recommending friends in online social networks, recommending products in e-commerce web sites^4–6^, and uncovering missing parts of social and biological networks^7–9^.

In link prediction, the central issue is how to predict the missing links effectively and accurately. Of the two aspects the accuracy problem is more fundamental from a theoretical point of view when computational cost is not a major concern. In literature this is termed as the “predictability problem”, i.e., to what extent can the missing or potential links be predicted, which to our knowledge was first proposed and investigated in^10^. Although the exact predictability can never be obtained due to the complexity and uncertainty intrinsic to real world networks, the practical predictability depends on the extent to which the available information can be exploited and the noise be eliminated. In principle, information is represented by something of regularity or consistency in the network structure when the network is dynamically changing due to, say, some evolution or perturbation processes. On the other hand, noise is represented by something irregular or inconsistent which is always unavoidable in real data sets. From this point of view, to improve link predictability, one should first find some proper description of the information and noise in the available data sets and then design some effective method accordingly.

In the context of complex networks which are usually described as graphs, the global topological information always lies in their adjacency matrices, in which nonzero entries denote links between corresponding nodes, while zero ones denote missing or nonexistent links. The adjacent matrix provides fundamental information for link prediction. And many existing link prediction algorithms are actually based on some kind of manipulations of the adjacent matrices or some of their variants. For example, the CN index^11^ of a node pair is the inner product of their corresponding rows of the adjacent matrix, and the RA index^11^ of some weighted adjacent matrix whose column sum is assigned as 1. These are local indices that have explicit physical meanings, while the Katz index^12^ uses the global information obtained from some series of the adjacent matrices. Motivated by these observations, new link prediction methods based on different kinds of manipulations of the adjacent matrices have been developed. Since the network structure can be well reflected by the eigenvectors of its adjacent matrix^13^, it is natural to make use of them in link prediction. Following the idea that the consistency in network structure can be represented by the eigenvectors of its adjacent matrices, the authors^10^ proposed a structural perturbation method (SPM) in which a new matrix was constructed for prediction by perturbing the eigenvalues of the adjacent matrix while fixing the eigenvectors. On the other hand, since the real data of complex networks all are always subjected to interfering noise, it is necessary to eliminate the noise to uncover the unobserved links. In^14^, by introducing the robust PCA technique, the authors developed a novel global information-based link prediction algorithm which decomposes the adjacent matrix into a low rank backbone structure and a sparse noise matrix.

Although the two aforementioned works have achieved considerable improvements compared with many existing methods, they still have some limitations. An important issue is that each of them only focuses on one of the two complementary aspects to accurate link prediction: information completion and noise reduction. Motivated by these observations, here we propose a novel link prediction method that combines these two complementary methods. That is, we first exploit the information from the adjacent matrix by some perturbation on its eigenvalues, then by the decomposition technique from robust PCA, we remove the sparse noise from the resulting matrix to reveal the backbone matrix for final prediction. Furthermore, for weighted directed networks which may have asymmetric adjacent matrices, we extract its symmetric part by introducing some new decomposition technique so that the original SPM is still applicable. Thus, our new method can be extended to weighted directed networks. Experimental studies indicate that this new method achieves considerable improvement when compared to each of the individual method in most of the networks.

## Results

Consider a weighted directed network *G*(*V*, *E*, *A*), where *V* = {*v*_1_, …, *v*_*n*_}, $E\subseteq V\times V$ are the set of nodes and links, respectively, and $A={[{a}_{ij}]}_{i,j=1}^{n}$ is the weighted directed adjacent matrix such that *a*_*ij*_ > 0 if node (*v*_*j*_, *v*_*i*_) $\in$ *E* and *a*_*ij*_ = 0 otherwise. If the network is undirected and unweighted, then *A* is a real symmetric matrix, i.e., *a*_*ij*_ = *a*_*ji*_ for each *i*, *j* = 1, 2, …, *n*. Otherwise, *A* may be an asymmetric matrix that has complex eigenvalues and is not diagonalizable. In such case the original SPM is not applicable. To test the accuracy of our new prediction algorithm, we randomly divide the link set *E* into a training set *E* ^*T*^ and a probe set *E* ^*P*^. Here *E* ^*T*^ is treated as known information or observed information, while *E* ^*P*^ is considered as the set of missing links. Obviously, ${E}^{T}\cap {E}^{P}=\varnothing$ and ${E}^{T}\cup {E}^{P}=E$. Our purpose is to try to predict the links in *E* ^*P*^ based on the information in *E* ^*T*^.

In the experiment, we first apply the perturbation procedure to *A* ^*T*^, which is the adjacent matrix of *G*(*V*, *E* ^*T*^). To implement the perturbation, we randomly select a fraction of links from *E* ^*T*^ to constitute the perturbation set Δ*E* ^*T*^, whose adjacent matrix Δ*A* ^*T*^ acts as the perturbation to *A* ^*T*^ − Δ*A* ^*T*^. As in^10^, in each prediction the final perturbed matrix ${\tilde{A}}_{T}$ is obtained by averaging over 10 independent selections of Δ*E* ^*T*^. Then we apply the robust PCA technique to ${\tilde{A}}_{T}$ to obtain the backbone structure ${\tilde{A}}_{B}$. In this procedure, the parameter *λ* for each network is chosen as the optimal value in the simulations. Particularly, based on some preliminary simulations, we found that is almost all cases, the optimal values of *λ* always fall in the interval (0, 0.4). Thus in the experiment, we simulate different values of *λ* from 0.01 to 0.39 at a step 0.01 and select the value that has the best performance. In the final prediction, we use the matrix ${\tilde{A}}_{B}$ as the score matrix, whose entries corresponding to the unconnected nodes are explained as their connection likelihood.

To measure the prediction accuracy of the algorithm, we use two standard metrics: *precision* ^15^ and *AUC* ^16^ which are defined as follows.

Given the ranking of the non-observed links according to their scores in descending order, if *L*_*r*_ of the top-*L* links, which are taken as the predicted ones, appear in the probe set, then *precision* = *L*_*r*_/*L*. In the experiment, we take *L* as the number of links in the probe set.

Given the ranking of the non-observed links, AUC is the probability that a randomly chosen missing link has a higher score than a randomly chosen nonexistent link. In the algorithmic implementation, instead of computing its exact value, AUC is usually approximated by the comparison of scores between node pairs randomly chosen from the set of missing links and of nonexistent links. If among *n* times of independent comparisons, there are *n*′ times the scores of the missing links are higher than those of the non-observed links, and *n*″ times they are the same, then

1 $$AUC=\frac{{n}^{{\prime} }+0.5{n}^{{\prime\prime}}}{n}$$

In the experiment, we test our algorithm on both undirected and directed networks from disparate fields. And we compared the results of our method with the original individual methods SPM and Low Rank (LR), as well as some other typical local indices including Common Neighbors(CN)^11^, Adamic-Adar (AA)^17^ and Resource Allocation(RA)^11^. The prediction results in precision and AUC are respectively presented in Tables 1 and 2 for undirected networks and in Tables 3 and 4 for directed networks, where for each network the result is obtained by averaging over 100 independent runs. Since the original SPM only applies to symmetric matrices, here the results for directed networks are obtained by the generalized one described in stage 1 of our method.

**Table 1:** The average predicting precision obtained by 100 independent runs on 19 real undirected networks. The training set contains 90% of total connections.

Network	*CN*	*AA*	*RA*	*SPM*	*LR*	*MPD*
Karate	0.085	0.13	0.144	0.125	0.125(0.23)	**0**.**175**(**0**.**22**)
Football	0.110	0.128	0.118	0.243	0.213(0.17)	**0**.**246**(**0**.**12**)
Dolphin	0.080	0.118	0.099	0.138	0.084(0.25)	**0**.**150**(**0**.**32**)
Everglades	0.142	0.164	0.177	0.573	0.556(0.20)	**0**.**605**(**0**.**15**)
WorldTrade	0.365	0.426	0.431	**0**.**486**	0.432(0.12)	**0**.**486**(**0**.**39**)
Macaca	0.495	0.528	0.510	0.719	**0**.**749**(**0**.**18**)	0.747(0.14)
FWM	0.111	0.122	0.127	0.540	**0**.**556**(**0**.**14**)	0.555(0.13)
BUP	0.126	0.193	**0**.**198**	0.184	0.103(0.19)	0.186(0.09)
WorldAdj	0.046	0.066	0.055	0.105	0.035(0.13)	**0**.**109**(**0**.**09**)
FWF	0.066	0.073	0.073	0.539	0.557(0.14)	**0**.**569**(**0**.**18**)
Jazz	0.474	0.523	0.538	0.649	0.608(0.13)	**0**.**650**(**0**.**24**)
Contact	0.564	0.574	0.575	0.622	0.631(0.10)	**0**.**633**(**0**.**11**)
C. elegans	0.083	0.108	0.105	0.170	0.126(0.10)	**0**.**177**(**0**.**12**)
USAir	0.369	0.402	0.465	0.264	0.200(0.09)	**0**.**467**(**0**.**18**)
INF	0.275	0.328	0.345	0.239	0.175(0.13)	**0**.**375**(**0**.**29**)
Metabolic	0.115	0.195	0.268	**0**.**350**	0.216(0.10)	0.348(0.26)
Email	0.124	**0**.**162**	0.146	0.154	0.076(0.16)	0.157(0.34)
PB	0.171	0.173	0.150	**0**.**543**	0.522(0.07)	0.236(0.10)
Yeast	0.142	0.177	0.269	0.546	0.525(0.14)	**0**.**564**(**0**.**24**)

The values in the brackets are the values of optimal parameters of the methods in the simulations. The highest precisions are shown in boldface.

**Table 2:** The average predicting AUC obtained by 100 independent runs on 19 real undirected networks.

Network	*CN*	*AA*	*RA*	*SPM*	*LR*	*MPD*
Karate	0.692	0.728	0.736	0.784	0.561(0.23)	**0**.**789**(**0**.**23**)
Football	0.679	0.682	0.677	0.800	0.610(0.18)	**0**.**828**(**0**.**12**)
Dolphin	0.786	0.789	0.786	0.773	0.604(0.26)	**0**.**840**(**0**.**30**)
Everglades	0.681	0.692	0.701	0.939	0.928(0.12)	**0**.**946**(**0**.**16**)
WorldTrade	0.868	0.889	0.907	0.934	0.861(0.12)	**0**.**935**(**0**.**33**)
Macaca	0.945	0.945	0.948	0.985	0.949(0.17)	**0**.**989**(**0**.**18**)
FWM	0.710	0.712	0.716	0.926	0.873(0.13)	**0**.**932**(**0**.**19**)
BUP	0.886	0.895	**0**.**898**	0.889	0.658(0.17)	0.897(0.36)
WorldAdj	0.679	0.676	0.675	0.718	0.539(0.14)	**0**.**758**(**0**.**06**)
FWF	0.610	0.608	0.612	0.950	0.853(0.14)	**0**.**951**(**0**.**28**)
Jazz	0.956	0.964	0.973	0.976	0.868(0.13)	**0**.**979**(**0**.**27**)
Contact	0.959	0.962	0.961	0.960	0.863(0.10)	**0**.**980**(**0**.**02**)
C. elegans	0.847	0.862	0.869	0.894	0.562(0.10)	**0**.**904**(**0**.**34**)
USAir	0.953	0.965	**0**.**972**	0.942	0.841(0.08)	0.950(0.18)
INF	0.942	0.945	**0**.**947**	0.943	0.754(0.13)	0.946(0.31)
Metabolic	0.922	0.955	**0**.**960**	0.931	0.595(0.10)	0.937(0.38)
Email	0.856	0.858	0.857	0.899	0.562(0.16)	**0**.**910**(**0**.**25**)
PB	0.923	0.929	0.929	0.926	0.656(0.07)	**0**.**965**(**0**.**05**)
Yeast	0.915	0.917	0.917	0.969	0.878(0.14)	**0**.**987**(**0**.**14**)

The training set contains 90% of total connections. The values in the brackets are the values of optimal parameters of the methods in the simulations. The highest precisions are shown in boldface.

**Table 3:** The average predicting precision obtained by 100 independent runs on 17 real directed networks.

Network	*CN*	*AA*	*RA*	*SPM*	*LR*	*MPD*
Bison	0.366	0.403	0.376	0.243	0.413(0.17)	**0**.**462**(**0**.**09**)
Cattle	0.154	0.209	0.228	0.140	0.168(0.19)	**0**.**246**(**0**.**11**)
Football	0.158	0.225	0.233	0.169	0.325(0.20)	**0**.**358**(**0**.**12**)
Gramdry	0.154	0.180	0.250	0.352	0.613(0.17)	**0**.**618**(**0**.**17**)
Gramwet	0.145	0.187	0.263	0.347	**0**.**584**(**0**.**18**)	**0**.**584**(**0**.**18**)
Cypdry	0.119	0.189	0.209	0.320	0.488(0.19)	**0**.**527**(**0**.**19**)
Cypwet	0.117	0.177	0.200	0.338	0.505(0.23)	**0**.**511**(**0**.**20**)
Mangdry	0.084	0.119	0.128	0.335	0.519(0.16)	**0**.**531**(**0**.**14**)
Mangwet	0.085	0.118	0.133	0.340	0.511(0.15)	**0**.**521**(**0**.**14**)
Polbooks	0.078	0.122	0.127	0.106	0.093(0.22)	**0**.**162**(**0**.**15**)
Baydry	0.058	0.083	0.096	0.368	0.560(0.16)	**0**.**568**(**0**.**15**)
Baywet	0.064	0.082	0.094	0.353	0.548(0.16)	**0**.**556**(**0**.**15**)
C. elegans	0.061	0.065	0.052	0.078	**0**.**125**(**0**.**16**)	0.119(0.18)
USAir	0.226	0.282	0.290	0.340	0.349(0.12)	**0**.**370**(**0**.**05**)
Email-Eu	0.182	0.215	0.228	0.131	0.234(0.08)	**0**.**264**(**0**.**07**)
PB	0.173	**0**.**193**	0.147	0.126	0.107(0.18)	0.092(0.17)
CollegeMsg	0.013	0.015	0.021	0.022	0.017(0.06)	**0**.**048**(**0**.**02**)

The training set contains 90% of total connections. The values in the brackets are the values of optimal parameters of the methods in the simulationsl. The highest precisions are emphasized by boldface.

**Table 4:** The average predicting AUC obtained by 100 independent runs on 17 real directed networks.

Network	*CN*	*AA*	*RA*	*SPM*	*LR*	*MPD*
Bison	0.798	**0**.**801**	0.794	0.714	0.770(0.16)	0.779(0.09)
Cattle	**0**.**809**	0.798	0.802	0.517	0.676(0.16)	0.800(0.14)
Football	0.966	0.964	**0**.**966**	0.791	0.725(0.22)	0.948(0.11)
Gramdry	0.750	0.761	0.757	0.757	0.924(0.14)	**0**.**956**(**0**.**13**)
Gramwet	0.752	0.763	0.760	0.748	0.935(0.14)	**0**.**963**(**0**.**15**)
Cypdry	0.783	0.781	0.780	0.822	0.868(0.14)	**0**.**951**(**0**.**13**)
Cypwet	0.783	0.783	0.779	0.832	0.867(0.14)	**0**.**952**(**0**.**11**)
Mangdry	0.761	0.775	0.783	0.771	0.887(0.12)	**0**.**945**(**0**.**12**)
Mangwet	0.769	0.783	0.787	0.785	0.893(0.12)	**0**.**951**(**0**.**09**)
Polbooks	0.903	0.891	0.903	0.876	0.553(0.22)	**0**.**918**(**0**.**38**)
Baydry	0.739	0.740	0.742	0.821	0.898(0.11)	**0**.**965**(**0**.**09**)
Baywet	0.742	0.746	0.751	0.822	0.902(0.12)	**0**.**956**(**0**.**10**)
C. elegans	0.805	0.809	0.812	0.902	0.593(0.16)	**0**.**903**(**0**.**07**)
USAir	0.966	0.964	**0**.**973**	0.955	0.833(0.10)	**0**.**973**(**0**.**22**)
Email-Eu	0.946	0.948	0.952	**0**.**966**	0.749(0.06)	0.962(0.02)
PB	0.928	0.916	0.927	0.943	0.669(0.04)	**0**.**961**(**0**.**02**)
CollegeMsg	0.735	0.747	0.755	**0**.**960**	0.546(0.24)	0.921(0.01)

The training set contains 90% of total connections. The values in the brackets are the values of optimal parameters of the methods. The highest precisions are emphasized by boldface.

From the experimental results, it can be seen that compared to each of the original individual methods, the new method achieves considerable improvements both in precision and in AUC. It gives the best results in most of the networks, especially in the directed ones. Even in the case it is not the best, it is still quite near the best in most cases. In some sparse networks such as CollegeMsg, the precision given by our method is more than two times of those given by others. It is also remarkable to note that in some cases, even when one or both of the two individual methods performs very poor, our method still works very well. This implies that the new method has not only a very competitive but also a very robust performance, which in our opinion is at least partly due to the complementary nature of SPM and LR and justifies the significance of their combination.

## Discussion

In this work, by generalizing and combining two previous link prediction methods which are of complementary nature, we propose a new algorithm for link prediction via perturbation and decomposition of the adjacent matrices of the networks. By exploiting the useful information and eliminating the interfering noise simultaneously, the new method takes advantage of both previous methods and robustly achieves considerable improvements on most of real world networks from disparate fields in experimental studies.

Beyond its competitive performances, the significance of this work, as well as that of those previous ones which it depends upon, is that they opened up a new direction for link prediction by directly manipulating the adjacent matrices as a whole. Compared to the classical methods such as similarity indices, link prediction algorithm in this new direction can no doubt take advantage of the rich tools and results available in matrix theory. And it is predictable that many new works in this direction will be done in the near future.

Since the new method is a combination of two existing methods, its computational complexity is roughly the summation of theirs. At stage 1, the time-consuming part is the computation of the eigenvalues and eigenvectors of the adjacent matrix whose complexity is *O*(*n* ^3^)^18^. At stage 2, it is the singular value decomposition (SVD) of the perturbed matrix whose complexity is *O*(*kn* ^2^)^14^ where *k* is the estimated rank of the matrix. Thus in summary, the computational complexity of the proposed algorithm is *O*(*n* ^3^).

Despite these advantages, the new method also faces some difficulties, among which the major one is how to determine the parameter *λ*. As many other parameterized methods, the parameter plays an important role in the performance of the algorithms, yet there is no explicit rule to determine its optimal value in advance. In the experiment we can choose for each network an optimal value based on the empirical simulations, yet this is not realistic for real-world applications. In that case, as in some learning algorithms, we can only obtain an estimated value of *λ* based on the training data. That is, we can divide the existent links into training set and probe set as we do in the experiment. Then we can obtain the “optimal” value of *λ* based on the simulations. Although this value generally is not the true optimal of *λ*, it should be at least an acceptable approximation, especially when the network is large enough. Moreover, to be safer, when it is possible, we can repeat this process many times and then determine an optimal value of *λ* based on the distribution of the outputs.

## Methods

Given a weighted directed network *G*(*V*, *E*, *A*), where *V*, *E*, and *A* are defined as before. In the following, we consider undirected networks as special cases of directed networks and will present the method in terms of directed networks in general.

In summary, our method consists of two stages: the perturbation stage and the decomposition one, which are described as follows.

### Stage 1: Structural perturbation

This stage can be divided into the following three steps.

Step 1: Preprocessing. Given the weight matrix *A* of a directed network, to apply the structural perturbation method to it, we first decompose it into the following two parts:

2 $$A={A}^{S}+{A}^{AS}$$

where *A* ^*S*^ = (*A* + *A* ^Τ^)/2 is the symmetric part of *A*, while *A* ^*AS*^ = (*A* − *A* ^Τ^)/2 is the antisymmetric part of *A*, with *A* ^Τ^ being the transpose of *A*. Intuitively, we explain the entries of *A* ^*S*^ as the average linking tendency between corresponding nodes, while the entries of *A* ^*AS*^ as the bias in the distribution of this tendency to the two links in opposite directions.

Step 2: Structural perturbation. Apply the structural perturbation method to *A* ^*S*^ as described in^10^, which for integrity will be briefly presented as follows. Since *A* ^*S*^ is a symmetric matrix, it can be written as

3 $${A}^{S}=\sum _{k=1}^{N}\,{\lambda }_{k}{x}_{k}{x}_{k}^{{\rm{T}}}$$

where *λ*_*k*_, *x*_*k*_, *k* = 1, 2, …, *n* are the eigenvalues and corresponding eigenvectors of *A* ^*S*^, respectively.

After some perturbation Δ*A* ^*S*^ to *A* ^*S*^, then the eigenvalue of *A* ^*S*^ + Δ*A* ^*S*^ will change to *λ*_*k*_ + Δ*λ*_*k*_ and its corresponding eigenvector to *x*_*k*_ + Δ*x*_*k*_. Thus we have

4 $$({A}^{S}+{\rm{\Delta }}{A}^{S})\,({x}_{k}+{\rm{\Delta }}{x}_{k})=({\lambda }_{k}+{\rm{\Delta }}{\lambda }_{k})\,({x}_{k}+{\rm{\Delta }}{x}_{k}),\quad k=1,\ldots ,n{\rm{.}}$$

Left-multiplying the eigenfunction by ${x}_{k}^{{\rm{T}}}$, and neglecting second-order terms ${\rm{\Delta }}{\lambda }_{k}{x}_{k}^{{\rm{T}}}{\rm{\Delta }}{x}_{k}$ and ${x}_{k}^{{\rm{T}}}{\rm{\Delta }}{A}^{S}{\rm{\Delta }}{x}_{k}$, we can obtain

5 $${\rm{\Delta }}{\lambda }_{k}\approx \frac{{x}_{k}^{{\rm{T}}}{\rm{\Delta }}{A}^{S}{x}_{k}}{{x}_{k}^{{\rm{T}}}{x}_{k}},\quad k=1,\ldots ,n{\rm{.}}$$

Fixing the eigenvectors and using the perturbed eigenvalues, we can obtain the perturbed matrix,

6 $${\tilde{A}}^{S}=\sum _{k=1}^{n}\,({\lambda }_{k}+{\rm{\Delta }}{\lambda }_{k}){x}_{k}{x}_{k}^{{\rm{T}}}.$$

Step 3: Postprocessing. Add the antisymmetric part of *A* to ${\tilde{A}}^{S}$ to get the final perturbed matrix:

7 $$\tilde{A}={\tilde{A}}^{S}+{A}^{AS}.$$

Here we fix the antisymmetric part of *A* based on the idea that the difference between the linking tendency of opposite directions are not changed obviously during the perturbation process.

### Stage 2: Noise reduction

In this stage we will remove the supposed noise from the perturbation matrix $\tilde{A}$ and recover the backbone structure for prediction. For this purpose we introduce the robust principal component analysis (robust PCA) as in^14^. For the sake of integrity we briefly present it in the following. If the network is highly regularly organized, then its backbone structure should have some low rank property, and the noise should be sparse. Thus we should decompose the matrix $\tilde{A}$ into two parts: a low rank part ${\tilde{A}}_{B}$ as the backbone structure and a sparse part ${\tilde{A}}_{N}$ as the noise. Mathematically, this can be transformed into the following optimization problem:

8 $$\mathop{{\rm{\min }}}\limits_{{\tilde{A}}_{B},{\tilde{A}}_{N}}\,{\rm{rank}}({\tilde{A}}_{B})+\gamma {\parallel {\tilde{A}}_{N}\parallel }_{0}\quad {\rm{subject}}\,{\rm{to}}\quad \tilde{A}={\tilde{A}}_{B}+{\tilde{A}}_{N},$$

where rank(·) denotes the rank of a matrix, ${\parallel \cdot \parallel }_{0}$ is the *l*_0_-norm of a matrix, and *γ* is the parameter that balances these two expressions. Since this is a highly nonconvex optimization problem that is hard to solve, we use some approximate solution based on robust PCA^19^, which is the solution of the following optimization problem:

9 $$\mathop{{\rm{\min }}}\limits_{{\tilde{A}}_{B},{\tilde{A}}_{N}}\,{\parallel {\tilde{A}}_{B}\parallel }_{* }+\lambda {\parallel {\tilde{A}}_{N}\parallel }_{1}\quad {\rm{subject}}\,{\rm{to}}\quad \tilde{A}={\tilde{A}}_{B}+{\tilde{A}}_{N},$$

where ${\parallel \cdot \parallel }_{* }$ is the nuclear norm of a matrix, ${\parallel \cdot \parallel }_{1}$ is the *l*_1_-norm, and *λ* is the parameter that balances the two expressions.

At last, we predict some missing or potential links based on the approximated backbone structure matrix ${\tilde{A}}_{B}$ as in^14^. That is, we take the entries in ${\tilde{A}}_{B}$ corresponding to the unobserved links as their similarity scores and sort them in a descending order. Then we select the top *L* links as our prediction result, where *L* is determined by some other rules.

## Data

For experimental studies, we have collected 36 real-world networks from disparate fields, including 19 undirected networks and 17 directed ones. These networks were carefully selected to cover a wide range of properties, including different sizes, average degrees, clustering coefficients, and heterogeneity indices. The basic topological features of the networks are summarized in Tables 5 and 6, respectively. A brief description of these networks are as follows:

**Table 5:** The basic topological features of 19 real undirected networks.

Network	|*V*|	|*E*|	*C*	*r*	〈*k*〉	〈*d*〉	*H*
Karate	34	78	0.5706	−0.4756	4.5882	2.4082	1.6933
Football	35	118	0.3390	−0.1763	6.7429	2.1227	1.4881
Dolphin	62	159	0.2590	−0.0436	5.1290	3.3570	1.3268
Everglades	69	880	0.5521	−0.2983	25.5072	1.6360	1.2746
WorldTrade	80	875	0.7525	−0.3918	21.8750	1.7241	1.5576
Macaca	94	1515	0.7736	−0.1506	32.2340	1.7712	1.2383
FWM	97	1446	0.4683	−0.1506	29.8144	1.6929	1.2656
BUP	105	441	0.4875	−0.1279	8.4000	3.0788	1.4207
WorldAdj	112	425	0.1728	−0.1293	7.5893	2.5356	1.8149
FWF	128	2075	0.3346	−0.1117	32.4219	1.7763	1.2370
Jazz	198	2742	0.6175	0.0202	27.6970	2.2350	1.3951
Contact	264	2108	0.6577	−0.4790	15.9697	2.3971	3.5461
C. elegans	297	2148	0.2924	−0.1632	14.4646	2.4553	1.8008
USAir	332	2126	0.6252	−0.2079	12.8072	2.7381	3.4639
INF	410	2765	0.4558	0.2258	13.4878	3.6309	1.3876
Metabolic	453	2025	0.6465	−0.2258	8.9404	2.6638	4.4850
Email	1133	5451	0.2202	0.0782	9.6222	3.6060	1.9421
PB	1222	16714	0.3203	−0.2213	27.3552	2.7375	2.9707
Yeast	2374	11693	0.3059	0.4538	9.8509	5.0932	3.4745

|*V*| and |*E*| represent the total numbers of nodes and links, respectively. *C* is the clustering coefficient^43^ and *r* denotes the assortative coefficient^21^. 〈*k*〉 is the average degree, and 〈*d*〉 is the average shortest distance. *H* is the degree heterogeneity denoted as *H* = 〈*k* ^2^〉/〈*k*〉.

**Table 6:** The basic topological features of 17 real directed networks.

Network	|*V*|	|*E*|	*C*	*r*	〈*k*〉	*H*
Bison	26	314	0.5099	4.5204	7.0769	1.3101
Cattle	28	217	0.2186	7.6487	0.8571	2.3333
Football	35	118	0.3390	−0.1763	6.7429	1.4881
Gramdry	69	879	0.5502	−0.2968	25.4783	1.2740
Gramwet	69	880	0.5521	−0.2983	25.5072	1.2746
Cypdry	71	618	0.4995	−0.2749	17.4085	1.4339
Cypwet	71	612	0.5004	−0.2773	17.2394	1.4369
Mangdry	97	1445	0.4608	−0.1519	29.7938	1.2613
Mangwet	97	1446	0.4683	−0.1506	29.8144	1.2656
Polbooks	105	441	0.4875	−0.1279	8.4000	1.4207
Baydry	128	2106	0.3346	−0.1044	32.9063	1.2307
Baywet	128	2075	0.3346	−0.1117	32.4219	1.2370
C. elegans	306	2345	0.1245	−0.2270	1.2876	2.7912
USAir	332	2126	0.0130	−0.2100	12.8070	4.9150
Email-Eu	1005	24929	0.2835	−0.0678	17.6418	2.7559
PB	1224	19087	0.1986	−0.2215	3.7876	6.7734
CollegeMsg	1899	59835	0.1685	0.3495	247.6388	18.5074

|*V*| and |*E*| represent the total numbers of nodes and links, respectively. *r* denotes the assortative coefficient. 〈*k*〉 is the average degree. *H* is the degree heterogeneity denoted as *H* = 〈*k* ^2^〉/〈*k*〉.

### Undirected network

- Karate^20^: The network of relationship among the members in the karate club.
- Football^21^: The network of American football games consisting of Division IA colleges during the regular season, Fall in 2000.
- Dolphin^22^: network of bottlenose dolphins living in Doubtful Sound (New Zealand).
- Everglades^23^: A network of foodweb in Everglades Graminoids during wet season.
- WorldTrade^24^: the network of miscellaneous manufactures of metal among 80 countries in 1994.
- Macaca^25^: cortical networks of the macaque monkey.
- FWM^26^: the food web in Mangrove Estuary during the wet season.
- BUP^27^: A network of political blogs.
- WorldAdj^28^: An adjacency network of common adjectives and nouns in the novel David Copperfield by Charles Dickens.
- FWF^29^: the network of predator-prey interactions in Florida Bay in the dry season.
- Jazz^30^: jazz musician network, the link denotes the relationship between two persons if they used to play together in the same band at least once.
- Contact^31^: a contact network between people measured by carried wireless devices.
- C. elegans^32^: A neural network of the nematode worm C. elegans compiled by D. Watts and S. Strogatz.
- USAir^23^: A network of US air transportation system, which contains 332 airports and 2126 airlines.
- INF^27^: A network of face-to-face contacts in an exhibition.
- Metabolic^32^: the metabolic network of the nematode worm C. elegans.
- Email^33^: the network of email interchanges between the members of the University of Rovira I Virgili.
- PB^34^: the network of hyperlinks between weblogs on US politics.
- Yeast^35^: the network of protein-protein interaction.

### Directed network

- Bison^36^: This directed network contains dominance between American bisons in 1972 on the National Bison Range in Moiese (Montana).
- Cattle^37^: This directed network contains dominance behaviours observed between dairy cattles at the Iberia Livestock Experiment Station in Jenerette, Louisiana.
- Football^38^: the network of American football games consisting of Division IA colleges during the regular season, Fall in 2000;
- Gramdry^39^: the network of predator-prey interactions in Everglades Graminoids in the dry season.
- Gramwet^39^: the network of predator-prey interactions in Everglades Graminoids in the wet season.
- Cypdry^39^: the network of predator-prey interactions in Cypress in the dry season.
- Cypwet^39^: the network of predator-prey interactions in Cypress in the wet season.
- Mangdry^39^: the network of predator-prey interactions in Mangrove Estuary in the dry season.
- Mangwet^39^: the network of predator-prey interactions in Mangrove Estuary in the wet season.
- Polbooks^40^: A network of books about US politics published around the time of the 2004 presidential election and sold by the online bookseller Amazon.com.
- Baydry^39^: the network of predator-prey interactions in Florida Bay in the dry season.
- Baywet^39^: the network of predator-prey interactions in Florida Bay in the wet season.
- C. elegans^32^: A neural network of the nematode worm C. elegans compiled by D. Watts and S. Strogatz;
- USAir^23^: A network of US air transportation system, which contains 332 airports and 2126 airlines;
- Email-Eu^41^: the network was generated using email data from a large European research institution.
- PB^34^: A directed network of hyperlinks between weblogs on US politics, recorded in 2005 by Adamic and Glance.
- CollegeMsg^42^: This dataset is comprised of private messages sent on an online social network at the University of California, Irvine.

## Benchmarks

For comparison, we introduce three benchmarks similarity indices based on structural information, including Common Neighbors(*CN*), Adamic-Adar Index(*AA*), and Resource Allocation Index(*RA*).

- Common Neighbors(*CN*). It supposes that two nodes are more likely to be connected if they have more common neighbors, so the number of their common neighbors can be regarded as a measurement of their similarity. Let Γ(*x*) denote the set of neighbors of *x*, |*Q*| denote the cardinality of the set *Q*, then *CN* is defined as
  10 $${S}_{xy}^{CN}=| {\rm{\Gamma }}(x)\cap {\rm{\Gamma }}(y)| .$$
- Adamic-Adar(*AA*). It can be seen as a refined *CN* index by assigning different weights to different nodes in the set of common neighbors. The larger degree of the common neighbor, the less weight it can contribute, then AA can be calculated by
  11 $${S}_{xy}^{AA}=\sum _{z\in {\rm{\Gamma }}(x)\cap {\rm{\Gamma }}(y)}\,\frac{1}{{\rm{log}}(| {\rm{\Gamma }}(z)| )}{\rm{.}}$$
- Resource Allocation(*RA*). It is similar to *AA*, but motivated by the resource allocation process on complex networks. It models the transmission of resources between two unconnected nodes through neighborhood nodes, then *RA* can be written as

12 $${S}_{xy}^{RA}=\sum _{z\in {\rm{\Gamma }}(x)\cap {\rm{\Gamma }}(y)}\,\frac{1}{| {\rm{\Gamma }}(z)| }{\rm{.}}$$
